# Factors affecting life satisfaction among retired older adults

**DOI:** 10.3389/fpubh.2025.1367638

**Published:** 2025-01-20

**Authors:** Minkyung Gu, Jakyung Yu, Sohyune Sok

**Affiliations:** ^1^Department of Nursing, College of Health Sciences, Daejin University, Pocheon-si, Republic of Korea; ^2^Department of Nursing, Graduate School, Kyung Hee University, Seoul, Republic of Korea; ^3^College of Nursing Science, Kyung Hee University, Seoul, Republic of Korea

**Keywords:** aged, retired, life satisfaction, health, self-efficacy, social activity, family relationships

## Abstract

**Background:**

The population of older adult people is rapidly increasing in South Korea. Consequently, older adult people’s life satisfaction, especially the retired older adult, is emerging as a social issue and not just an individual problem.

**Objective:**

This study aimed to examine and identify the factors affecting the life satisfaction of the retired older adult.

**Methods:**

A cross-sectional descriptive design was employed. The sample included 149 retired older adults living in Gyeonggi or Seoul, South Korea. The data included the general characteristics of study participants and the level of their life satisfaction, health conservation, self-efficacy, and social activity. The data was collected from January to February 2020 and analyzed using SPSS PC+ version 23.0.

**Results:**

The factors affecting life satisfaction in retired older adults were health conservation (*β* = 0.28, *p* < 0.001), self-efficacy (*β* = 0.26, *p* = 0.001), marital status (β = 0.24, *p* = 0.001), social activity (β = 0.18, *p* = 0.018), and family relationships (β = −0.15, *p* = 0.048). The explanatory power of the final regression model was 59%.

**Conclusion:**

The study suggests that to improve the life satisfaction of retired older adults, their health conservation, self-efficacy, social activity, and family relationships should be increased, and married status with spouse needs to be considered. Most importantly, health professionals need to pay attention to the factors that improve the life satisfaction of retired older adults in community fields.

## Introduction

The recent rapid increase in the aging population has brought about changes in the economic and social structure caused by industrialization. Super-aged society is expected as the older adult population aged 65 years or older will account for 20.8% of the total population by 2026 (1, 2). In 2047, 49.6% of all households are expected to be older adult households. Specifically, the older adult population in South Korea is expected to increase from 8.15 million in 2020 to 13.06 million in 2030, and 17.47 million in 2070. The proportion of the older adult population in South Korea was 15.7% of the total population in 2020, which was a low figure in Organization for Economic Co-operation and Development (OECD) countries but is expected to increase to the highest level in 2070 (46.4%) (3), which is triggering various changes in the status and roles of the older adult related to social activities in Korean society (3, 4). These social phenomena have recognized the older adult as independent members of society, shifting away from looking at the older adult from a declining point of view (5, 6).

In South Korea, people over the age of 65 are generally considered older adult. In addition to the deterioration of physical functions, the older adult may experience psychological changes, loss of social roles, and aging, a gradual change process toward death (2, 7, 8). Compared to other generations, the level of life satisfaction of the older adult persistently may drop due to chronic diseases, a decrease in economic ability due to retirement, and a decrease in social roles (9, 10). As a result, the self-esteem of the older adult decreases, which may lead to psychological alienation and loneliness (2, 10, 11).

It is difficult for the older adult to change easily the life patterns and consumption culture that they have been accustomed to despite the decrease in income as a result of retirement. They want to pursue life satisfaction by engaging in economic activities rather than reducing the scale of consumption and changing life patterns (4, 12). Therefore, it can be assumed that life satisfaction for the older adult is crucial in an aging society, as an increase in the older adult population is expected.

The average retirement age in South Korea is 61.0 years, while the proportion of retirees aged 60 years or older is only 8.1% of all retirees. Comparing the official retirement age and actual retirement age of OECD member countries from 2013 to 2018, Korea showed the largest gap between the official and actual retirement age among OECD member countries (13). While the official retirement age of Korean older adult was 61, the actual retirement age was 72.3, which was a large difference from the actual average retirement age of OECD member countries (65 years old), Luxembourg (61 years old), and Sweden and the United States (mid-60 years old). Therefore, it can be seen that Korean older adult people are working for more than 10.3 years after the national pension start age of 62, that is, the official retirement age (13). In South Korea, the most common reason the older adult work for a long time even when they are old is to cover the cost of living, because social security systems, such as personal savings, retirement pay, and national pension, are insufficient to maintain a livelihood in old age. Therefore, most seniors have to live for more than 30 years on reduced income after retirement (6, 8). Furthermore, it was found that Korean older adult retirees want to work even in old age. Judging from the fact that the overwhelming majority of reasons are ‘because they need money financially,’ this can be seen as supporting the need for swift implementation of economic support policies and interventions by the state and government (6, 7).

A retired older adult is a person who has worked in a field with acquired knowledge and skills for a considerable amount of time (10). As life becomes abundant and the economic level increases, the retired older adult continues to pursue their social activity in relation to role behaviors and status (6, 10, 12). However, there is a significant reduction in the social activities that can be called jobs of retired seniors. In particular, retired older adults may perceive themselves as useless and helpless because they are unemployed, making them live in isolation and eventually become psychologically weak and dependent (6, 14). They may only show their efforts to fill their daily lives with increasing dependence on their family members (15, 16). These problems may have also been found to negatively affect the health preservation of the retired older adult (6, 17).

On the other hand, social activities for the retired older adult positively impact physical and social health preservation by helping them maintain a normal life with economic stability and induce adaptation to a newly changed living environment through exchanges with various people (7, 18). Social activity leads to the value realization of life for self-realization and self-efficacy neglected in the youth of retired older adults, which results in psychological well-being and life satisfaction (19, 20). In addition, social activities for the retired older adult can relieve stress and exude enjoyment and new challenges (12, 21). For the retired older adult, social activity is a necessary condition to give them a new lease on life, which can be a key factor in their successful aging (4, 14, 22).

The level of life satisfaction of the older adult may be related to sociodemographic information, such as the occupation before retirement, years of service before retirement, and length of retirement (20, 22), but, in general, the level of life satisfaction among the older adult is representatively explained from the perspective of deprivation of social roles and activities (23, 24). The older adult can recognize themselves as social beings and achieve self-reflection while performing their roles. Thus, it is argued that the social being arises from interactions with the outside world, so new roles and activities must be actively performed (24). Therefore, it can be said that how much the older adult must find new roles and engage in activities is closely related to their life satisfaction (23).

**Figure 1:**
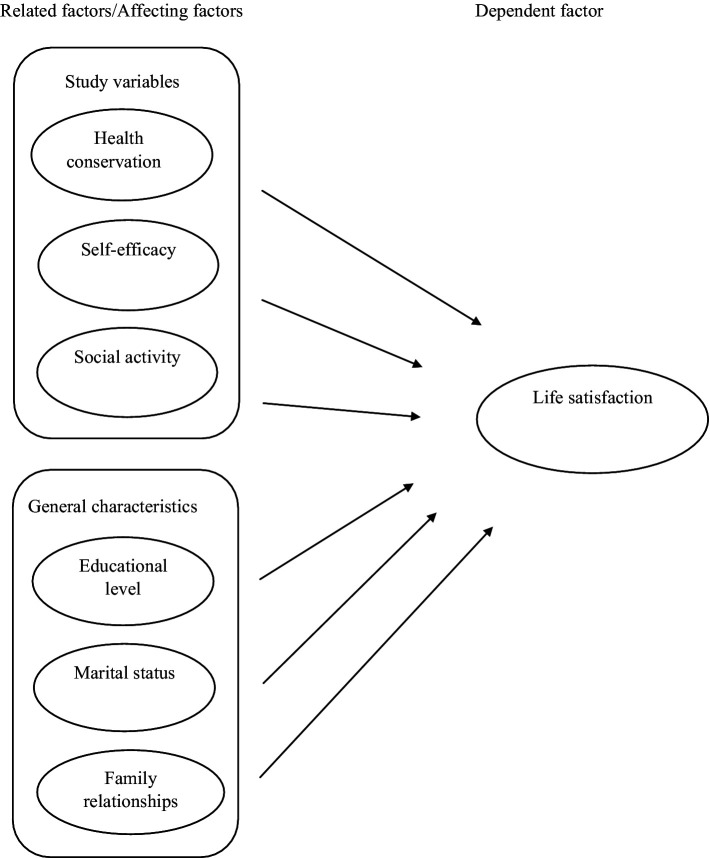
Conceptual framework.

Figure 1 shows the conceptual framework in this study. The factors affecting the life satisfaction of retired older adults used in this study were health conservation, self-efficacy, social activity, educational level, marital status, and family relationships. These independent factors affect the life satisfaction of the retired older adult and are categorized by study variables (health conservation, self-efficacy, social activity) and general characteristics (educational level, marital status, and family relationships). They were selected as a result of the findings of previous research (5, 14, 22).

The recent increase in the retirement of the older adult has led to a decrease in economic income, loss of social roles, and decrease in physical and mental health due to the aging process, thereby lowering their self-esteem and acting as a cause of disease (24–26). Furthermore, the retired older adult was found to have a lower positive perception of physical, social, and psychological adaptation as a result of aging than the general retired older adult people, which negatively affected their relationships (7, 24). Therefore, a specific intervention plan is needed for the retired older adult to encourage them to voluntarily participate in social activities and yield a more settled and significant life. Hence, this study is considered necessary for the appropriate health promotion of the retired older adult in South Korea and may conduce to the exploitation of nursing interventions that reflect empirical characteristics of the retired older adult.

The study aimed to examine the factors affecting life satisfaction among the retired older adult. Specifically, the research intended to (1) identify the factors related to the life satisfaction of the selected retired older adult in South Korea and (2) examine the predictive effect or the association of the general characteristics of study participants with life satisfaction.

## Methods

### Study population

**Figure 2:**
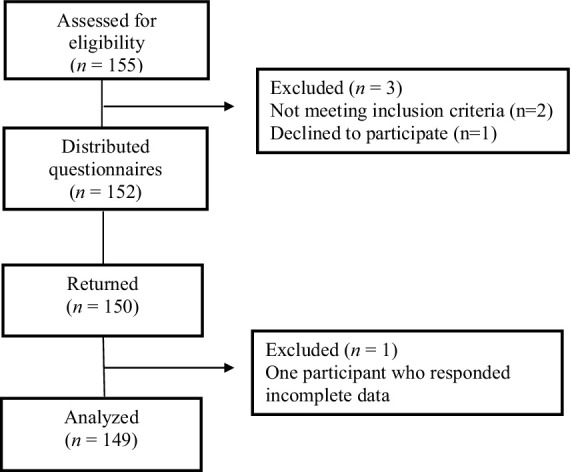
Flowchart of subject progress.

A cross-sectional descriptive design was used to measure and verify the factors affecting life satisfaction among the retired older adult in this study. The participants, chosen through convenience sampling, were 149 retired older adults living in Gyeonggi or Seoul, South Korea. They were recruited with the help of the community center personnel. Eligibility criteria were as follows: 65 years of age or older, those who agreed to participate in this study, those who understood the study’s purpose, those who had the cognitive ability to respond, and those who could understand each other in Korean. On the other hand, those with a cognitive function score (Mini-Mental State Examination, MMSE) of 20 or less and current acute illnesses were excluded from the study. Of the 152 questionnaires, 150 (98.7%) were retrieved. However, only 149 questionnaires were used in this study because one questionnaire had incomplete data (Figure 2). G power 3 analysis software was operated for sample size adequacy. The estimation using an alpha level of 0.05, medium effect size of 0.15, and power of 0.95 (27) was presented to show the need for a minimum of 138 survey answers. Therefore, the study’s intended sample size was satisfied.

The majority of the participants were male (male: 61.9%, female: 38.1%). The mean age was 66.88 years old, and the age group from 67 to 69 years old was the biggest age group at 63.1%. As for the education level, most were middle or high school graduates, showing a moderate education level. For the marital status, 85.2% were married. The monthly income of the retired older adult before retirement was 2 million won to 3 million won, 34.9% and 1 million won to 2 million won 30.9%, respectively. Regarding family relationship satisfaction, most participants (94%) answered “moderate” or “good.” As for the economic status, 94% answered “moderate” or “good” (Table 1).

**Table 1:** General characteristics of the study participants.

Characteristics	*n*	%
Gender		
Male	88	61.9
Female	61	38.1
Age (year)		
65 to <67	42	28.2
67 to <69	94	63.1
≥ 69	13	8.7
Educational level		
≤ Elementary school	4	2.7
Middle, High school	100	67.1
≥ College	45	30.2
Marital status		
Married	127	85.2
Bereaved	8	5.4
Divorced	11	7.4
Single	3	2.0
Family relationships		
Good	79	53.1
Moderate	61	40.9
Bad	9	6.0
Monthly income (USD)		
< 700	16	10.7
700 to <1,400	46	30.9
1,400 to <2,100	52	34.9
≥ 2,100	35	23.5
Economic status		
Good	53	35.6
Moderate	87	58.4
Bad	9	6.0

### Measurements

#### General characteristics of study participants

The participant’s general characteristic variables included seven items, including gender, age, educational level, marital status, family relationships, monthly income, and economic status, according to the literature review (5, 7, 14, 22, 24–26). The economic status was divided into good, moderate, and bad according to the person’s perception of the economy and was answered using self-report.

#### Life satisfaction

The life satisfaction scale for the life satisfaction of the older adult developed by Neugarten et al. (28) and translated by Lee (29) was used in this study. This included 14 items, namely, self-esteem, achievement, attitude toward aging, and satisfaction in a real relationship. A five-point Likert scale was used, with a score ranging from 14 to 70 points, where a higher score means higher life satisfaction. As regards the Korean version of the life satisfaction scale, at the time of the development of the items of life satisfaction (28), Cronbach’s *α* = 0.81. During the actual study, Cronbach’s α = 0.91.

#### Health conservation

The health conservation scale developed by Sung (30) was employed to find out the subject’s thoughts on health conservation. The tool has 37 items, where health conservation of personal integrity covers 14 items (e.g., I eat regularly, I cannot sleep well at night, I’m tired, fecal and urine excretion are smooth, etc.), energy health conservation related to activities of daily life comprised eight items (e.g., I usually exercise, there is no problem in seeing objects or hearing sounds, I have slow body movement, etc.), health preservation of structural integrity, such as thoughts have eight items (e.g., I express interest and love for others, I respect myself, I make important decisions for myself, etc.), and health conservation of social integrity related to activities cover seven items (e.g., I participate well in social gatherings, I do community service, I enjoy hobbies, etc.). A four-point Likert scale was used, with a score ranging from 37 to 148 points, where a higher score means higher health conservation. The health conservation scale, validated as Cronbach’s *α* = 0.94 at the time of the development (30), in the present study, the Cronbach’s alpha coefficients for health conservation of personal integrity (14 items), energy health conservation related to daily life (8 items), health preservation of structural integrity, such as thoughts (8 items), health conservation of social integrity related to activities (7 items) subscales were 0.92, 0.94, 0.93, and 0.93, respectively. In this study, the total Cronbach’s *α* = 0.93.

#### Self-efficacy

The self-efficacy scale developed by Sherer et al. (31) and translated by Hong (32) was used to determine the respondents’ self-efficacy. The instrument comprised 23 items, with 17 general self-efficacy items and six social self-efficacy items. A five-point Likert scale was used, with a score ranging from 23 to 115 points, indicating that the higher the score, the higher the self-efficacy. In the present study, the Cronbach’s alpha coefficients for general self-efficacy (17 items) and social self-efficacy (6 items) subscales were 0.85 and 0.87, respectively. At the time of the translation to the Korean version (32), Cronbach’s *α* = 0.86, and reliabilities of the scale in this study, the total Cronbach’s α = 0.86.

#### Social activity

The social activity scale developed by Jang (33) was used to investigate the meaning given to social activity by the older adult. Social activity is defined as positive satisfaction and purpose in the life of the older adult. The instrument included 14 items to identify the older adult’s self-esteem, economic benefits, attractiveness of social activity, social belonging, and reward for social activity. A seven-point Likert scale was used with a score ranging from 14 to 98 points, presenting that the higher the score, the higher the social activity for the older adult. At the time of the development (33), Cronbach’s *α* = 0.90, and the reliability of this study was Cronbach’s α = 0.77.

#### Data collection

The data collection was conducted from January to February 2020. The authors met the preliminary retired older adult participants to inform them about the study’s process, aims, questionnaire, and details. Written informed consents were retrieved from the retired older adult participants who had voluntary participation. They were provided with the questionnaires, which they completed for around 20–25 min. Then, the accomplished questionnaires, which were answered using self-reporting, were collected by the researchers.

#### Ethical considerations

The Institutional Review Board in Daejin University approved this study (IRB no. 1040656-201912-SB-02-13, approval date February 9, 2020). The participants were informed about voluntary participation and were available to withdraw at any time. They were also informed about anonymity and confidentiality and that after the study was completed, all data would be shattered by machine.

#### Data analysis

Descriptive statistics was applied to evaluate the general characteristics of the study participants and levels of study variables. Specifically, SPSS PC+ version 23.0 statistical software program (IBM Inc., Armonk, NY, USA) was used to analyze the data of the study. The normality of the data distribution was examined using the Shapiro–Wilk test. The independent t-test, ANOVA (F test), and Scheffe *post hoc* test analyzed the differences in life satisfaction according to the general characteristics of the participants. Pearson’s correlation coefficient interpreted the correlations between life satisfaction and related factors. Among the general characteristics of the study participants, statistics on nominal and continuous variables was obtained through linear regression analysis. Lastly, the hierarchical stepwise multiple regression was analyzed to examine the factors affecting life satisfaction. A *p*-value less than 0.05 was considered the statistically significant level.

## Results

### Levels of life satisfaction, health conservation, self-efficacy, and social activity

The mean score for life satisfaction was 57.23, which indicates a high life satisfaction when compared to the median value (42 points) of the score range (14–70). The mean score of health conservation was 112.60, which suggests high health conservation when compared to the median value (92.5 points) of the score range (37–148). Their mean score for self-efficacy was 75.64, implying a high self-efficacy when compared to the median value (69 points) of the score range (23–115). However, the mean score for social activity was 48.96, which infers a low social activity when compared to the median value (56 points) of the score range (14–98). The findings illustrate that study participants had a low mean score only for social activity but high mean scores for all the other study variables (Table 2).

**Table 2:** Levels of life satisfaction, health conservation, self-efficacy, and social activity.

Variables	Mean ± SD	Range point	Min	Max	Interquartile range (IQR)
Life satisfaction	57.23 ± 9.25	14–70	33.00	70.00	4
Health conservation	112.60 ± 20.32	37–148	72.00	167.00	4
Self-efficacy	75.64 ± 10.19	23–115	56.00	99.00	3
Social activity	48.96 ± 7.31	14–98	33.00	70.00	2

### Differences in life satisfaction according to the general characteristics of the study participants

There were differences in the mean scores of life satisfaction, in some of the participant characteristics related to the educational level (*F* = 4.89, *p* = 0.009), marital status (*F* = 3.60, *p* = 0.015), and family relationships (*F* = 6.82, *p* = 0.001). The results show that the higher the education level, married status, and family relationships, the higher the life satisfaction of the retired older adult (Table 3).

**Table 3:** Differences of life satisfaction according to the general characteristics of the study participants.

Characteristics	Mean ± SD	Independent t-test *Scheffe* post or F test (*P*) hoc test
Gender		
Male	46.88 ± 5.38	1.67 (0.364)
Female	42.32 ± 3.25	
Age (year)		
65 to <67	47.79 ± 9.02	
67 to <69	49.72 ± 6.57	0.72 (0.583)
≥ 69	47.23 ± 7.19	
Educational level		
≤ Elementary school^a^	44.75 ± 1.71	
Middle, High school^b^	51.62 ± 6.71	4.89 (0.009*) a.c < b
≥ College^c^	47.93 ± 7.40	
Marital status		
Married^a^	60.33 ± 11.55	
Bereaved^b^	48.59 ± 7.24	3.60 (0.015*) a > b,c,d
Divorced^c^	47.36 ± 5.24	
Single^d^	52.75 ± 5.47	
Family relationships		
Good^a^	50.89 ± 6.97	
Moderate^b^	47.11 ± 7.52	6.82 (0.001*) a > b,c
Bad^c^	44.56 ± 2.40	
Monthly income (USD)		
< 700	47.31 ± 5.50	
700 to <1,400	48.90 ± 5.17	0.36 (0.784)
1,400 to <2,100	49.48 ± 8.55	
≥ 2,100	49.03 ± 8.51	
Economic status		
Good	47.70 ± 7.91	
Moderate	49.87 ± 7.02	1.65 (0.195)
Bad	47.56 ± 5.39	

*P < 0.05.

### Correlations between life satisfaction and factors related to it

Life satisfaction had significant, positive relations with health conservation (*r* = 0.36, *p* < 0.001), self-efficacy (*r* = 0.46, *p* < 0.001), and social activity (*r* = 0.30, *p* < 0.016).

### Factors affecting life satisfaction

The assumptions of the regression model were tested as follows: After examining the residuals, homoscedasticity was confirmed. To verify the independence of the residuals, the autocorrelation of the error was tested with Durbin-Watson. As a result, the test statistic was 1.60, which is 1.59 to 1.76, satisfying all regression assumptions regardless of autocorrelation. Also, the multicollinearity tolerance was more than 0.10 from 0.72 to 0.97, and the coefficient of variance expansion (variance inflation factor, VIF) was from 1.03 to 1.39. In this study, the VIF value was not greater than 10, indicating that all variables had no multicollinearity problem.

The factors associated with life satisfaction in retired older adults were analyzed using the hierarchical stepwise multiple regression analysis. Consequently, the first-stage regression model with the general characteristics of the retired older adult was statistically significant (*F* = 5.14, *p* < 0.001). The variables that were statistically significant in the first stage were educational level (*β* = 0.17, *p* = 0.030), marital status (β = 0.22, *p* = 0.005), and family relationship (*β* = −0.32, *p* < 0.001). In the first stage, 39% of explanatory power was presented. In the second stage, the main variables, such as health conservation, self-efficacy, and social activity, were inputted. The second-stage regression model was also statistically significant in this study (*F* = 9.90, *p* < 0.001). The statistically significant variables in the second-stage regression model with the general characteristics of the retired older adult and main variables were marital status (*β* = 0.24, *p* = 0.001), health conservation (β = 0.28, *p* < 0.001), self-efficacy (β = 0.26, *p* = 0.001), social activity (β = 0.18, *p* = 0.018), and family relationships (β = −0.15, *p* = 0.048). The explanatory power of the second-stage regression model increased by 20% compared to the first stage. In this study, the most important variable affecting life satisfaction in retired older adults was health conservation, followed by self-efficacy, marital status, social activity, and family relationships. In the final stage, 59% of the explanatory power was presented (Table 4).

**Table 4:** Factors affecting life satisfaction.

Variables	Model 1						
	B	S.E	β	*t*	*P*	95% CI	
						Lower	Upper
Gender	2.01	1.98	0.04	1.21	0.218	−1.09	1.88
Age	−0.52	0.44	−0.09	−1.18	0.242	−1.01	1.11
Educational level	2.36	1.08	0.17	2.20	0.030*	−2.06	3.20
Marital status	2.47	0.88	0.22	2.82	0.005*	−0.52	3.28
Family relationships	−3.88	0.95	−0.32	−4.09	< 0.001*	−6.37	−1.64
Monthly income	−0.68	2.02	−0.23	−0.34	0.737	−4.64	4.33
Economic status	1.15	1.06	0.09	1.08	0.281	−2.08	2.91
R^2^ = 0.42, Adjusted R^2^ = 0.39, F = 5.14, *p* < 0.001*							

Variables	Model 2						
	B	S.E	β	t	*P*	95% CI	
						Lower	Upper
Gender	0.97	0.86	0.12	2.34	0.187	−0.72	0.33
Age	−0.25	0.39	−0.05	−0.65	0.516	−0.85	0.78
Educational level	1.92	1.00	0.14	1.92	0.057	−1.27	2.92
Marital status	2.71	0.76	0.24	3.55	0.001*	−0.41	2.50
Family relationships	−1.75	0.90	−0.15	−1.94	0.048*	−2.50	1.41
Monthly income	0.44	1.79	0.02	0.25	0.804	−1.69	5.24
Economic status	1.45	0.93	0.11	1.55	0.122	−1.31	2.53
Health conservation,	0.10	0.03	0.28	3.93	< 0.001*	0.03	0.13
Self-efficacy	0.18	0.06	0.26	3.28	0.001*	0.16	0.40
Social activity	0.14	0.06	0.18	2.39	0.018*	0.10	0.35
R^2^ = 0.63, Adjusted R^2^ = 0.59, *F* = 9.90, *p* < 0.001*							

Dummy variable: Educational level (Middle, High school, ≥ College), Marital status (Married), Family relationships (Good); CI = Confidence Interval. *p < 0.05.

## Discussion

The education level of most retired older adults who participated in this study was middle or higher school, followed by college. This was analogous to the findings of Sung et al. (6) confirming health conservation according to the education level of retired older adults living in big cities. Considering the educational level of retired older adults, it is imperative to broaden the customized education for the promotion of older adult health by reflecting on past social characteristics and encouraging them to actively participate in learning (2, 12, 34).

Additionally, the participants who were married or had a smooth family relationship were found to have a higher influence on life satisfaction. This finding is consistent with a study conducted in two major cities (Seoul and Incheon) (6). The results indicating that strong family support helps improve the older adult’s social activities and positively influences their health status, as it can be the recovery resource based on a sense of trust, is higher when the older adult’s family relationship is smooth (7, 16). This result is parallel with the findings confirmed by Lee and Kim (35) and Sung et al. (6), who carried out studies on the older adult living in a large city, which improves their perspective on the meaning of life, positively affecting their daily life. It therefore suggests that it is essential to help retired older adults pursue the meaning of life and allow opportunities for reflection regarding old age by forming positive family relationships and social interactions with others (4, 10). Above all, unlike the younger generation today, retired seniors have lived a life of service to their families, including spouses and children, and social organizations rather than to themselves. Retired seniors have often shown a submissive and passive attitude while pursuing social activities. In order for retired seniors to be productive and active socially, it is necessary to enrich their lives in old age and create a social culture that allows them to take the initiative (13, 17).

The correlations between the life satisfaction of the retired older adult and the study variables imply that life satisfaction was positively correlated with health conservation, self-efficacy, and social activity. This is analogous to the results of previous studies that revealed how physical activities related to work have a constant good effect on the retired older adult’s health status (4, 21, 22), and the pursuit of social activity of the retired older adult brings them emotional stability and life satisfaction (12, 20). Therefore, it is vital to encourage the retired older adult to develop a positive attitude toward independent life using direct and indirect support systems in the community and actively participate in community activities by increasing social activity programs for the retired older adult. This means that there is a need to organize health-related social activities for retired older adult people into more specific and practical content. In particular, before becoming older adult and retiring, it is necessary to seriously consider how to spend the rest of one’s life in adulthood or middle age (13, 17).

This study covered the following factors affecting the life satisfaction of retired older adults: health conservation, self-efficacy, marital status, social activity, and family relationships. This scope of factors supports the study results of Sung et al. (6) and Park and Oh (36), who claimed that the economic and social activities of the older adult ensure independence, thereby aiding their overall health and promoting self-realization by increasing their self-efficacy. These results also strengthen the conceptual framework used in this study. Furthermore, most of the previous studies support the research result that, regardless of the level of education, voluntary participation in work rather than simply providing livelihoods in old age, can influence the level of life satisfaction of the older adult (2, 16, 23). Therefore, it is necessary to support economic activities suitable for the older adult and increase their self-efficacy by creating different types of jobs. However, this is in contrast to the research results showing that the lower the level of education, the higher the meaning given to work, and thus the higher the life satisfaction (35), so it is necessary to conduct additional research by dividing the educational level of retired older adult people in detail while ensuring homogeneity in the future.

This study is significant because it identifies factors affecting the level of life satisfaction of the retired older adult in South Korea, underscores the need for a social support system in the local community, and prepares fundamental data for an intervention program that can help improve the emotional well-being of the retired older adult. Furthermore, understanding this study will lead to higher quality standards of care in the community. In addition, it will contribute to the expansion of international literature and follow-up studies on the retired older adult’s condition.

### Implications for practice, policy, and research

Based on the study’s results, healthcare professionals should pay attention to the influencing factors for improving the life satisfaction of the retired older adult in the community. The findings of this study can be used to develop interventions or strategies that will improve the life satisfaction of the retired older adults. In addition, further qualitative research is recommended to understand and analyze the life satisfaction of the retired older adult based on their perspective. Repetitive or longitudinal research on additional variables associated with the life satisfaction of retired older adult is also proposed. Importantly, it is necessary to create a vocational participation program considering the characteristics of the retired older adult’s past social activities and conduct an experimental study to determine its effectiveness. The strengths of this study are the diversity of variables and the unique setting of the subjects, which is retired older adult people, and its differentiation from previous studies. This study will be helpful in contributing to the promotion of health among the older adult.

### Limitations

This study was cross-sectional descriptive design. Inferences about casual relationships of variables may not be made. Also, all variables were measured using self-reported measures. Recall bias is plausible. This study included only retired older adults aged 65 years or older, which is a small sample size in some regions of South Korea. Therefore, due to these limitations of this study, generalizations should be treated with caution.

## Conclusion

In conclusion, for the retired older adult, life satisfaction was influenced by health conservation, self-efficacy, marital status, social activity, and family relationships. Various social activities for the older adult can help them form a healthy body and a positive mental state to enjoy the rest of their lives more productively and stably (10, 17, 19). Hence, the main task of public health for the retired older adult is to help them find jobs as they feel rewarded for their social activity and, importantly, encourage them to become active by participating in different health-related intervention programs.

## Data Availability

The raw data supporting the conclusions of this article will be made available by the authors, without undue reservation.
